# Sharp injuries: a cross-sectional study among health care workers in a provincial teaching hospital in China

**DOI:** 10.1186/s12199-017-0691-y

**Published:** 2018-01-10

**Authors:** Zhuo Cui, Jingrui Zhu, Xiangjun Zhang, Bairu Wang, Xiaojun Li

**Affiliations:** 1 Department of Infection Control, First Affiliated Hospital of Bengbu Medical College, 287 Changhuai Road, Bengbu, 233004 People’s Republic of China; 2 Department of Medical Affairs, First Affiliated Hospital of Bengbu Medical College, 287 Changhuai Road, Bengbu, 233004 People’s Republic of China

**Keywords:** Blood-borne diseases, Healthcare workers, Incidence, Sharp injuries, Underreporting

## Abstract

**Background:**

The objectives of this study are to investigate the incidence and reporting behavior of sharp injuries among healthcare workers (HCWs) and identify the risk factors associated with these injuries.

**Methods:**

A cross-sectional survey was conducted in February 2017 in a provincial teaching hospital in China. Data were collected from 901 HCWs using a self-administered questionnaire which included demographic information, experience, and reporting behavior of sharp injuries. Stepwise logistical regression was used to analyze the risk factors.

**Results:**

HCWs (248 [27.5%]) had sustained a sharp injury in the previous year. Factors including seniority, job category, title, education, department, and training programs were associated with the occurrence of sharp injuries. According to the stepwise logistical regression, seniority, and training programs were the risk factors associated with the occurrence of sharp injuries. Of 248 sharp injuries, 130 HCWs were exposed to blood. Only 44 (33.9%) HCWs reported their injuries to the concerned body. The main reasons for not reporting the sharp injuries were as follows: perception that the extent of the injury was light (30.2%), having antibodies (27.9%), and unaware of injury (16.3%).

**Conclusions:**

Sharp injuries in the studied hospital were common and were likely to be underreported. Therefore, an effective reporting system and sufficient education on occupational safety should be implemented by the relevant institutions. Moreover, it is important to take effective measures to manage sharp injuries in HCWs and provide guidance for their prevention.

## Background

Sharp injuries are defined as accidental skin penetrating stab wounds caused by hollow-bore needles such as hypodermic needles, blood-bore needles, catheter stylets, and needles used to connect parts of a delivery system. Sharp injuries are the most common occupational injuries among healthcare workers (HCWs). HCWs are especially prone to sharp injuries during their work periods and infection after exposure [1]. The World Health Organization estimates that 1 in 10 HCWs worldwide sustain a sharp injury each year [2]. Sharp instruments contaminated by blood-borne pathogens are the main factors in occupational exposure to blood-borne diseases among HCWs. They are at risk of infection due to blood-borne viruses including hepatitis B virus (HBV), hepatitis C virus (HCV), and human immunodeficiency virus (HIV) [3, 4]. Globally, approximately 66,000 HBV, 16,000 HCV, and 1000 HIV infections occurred in HCWs due to sharp injuries in 2000 [5]. The estimated annual costs for tests and the treatment of sharp injuries vary from $6.1 million in France to $118–591 million in the USA [6].

Reporting of sharp injuries is important for treatment and prevention. For the injured person, injury reporting prompts evaluation for post-exposure prophylaxis, allows early detection of seroconversion and helps to decrease anxiety. Furthermore, injury reporting allows identification of hazardous devices or procedures and so diminishes the risk of future injuries [7]. The underreporting of sharp injuries by employees has been documented in various studies. The magnitude of underreporting is 22 to 99% [8]. Due to the low reporting rate, post-exposure prophylactic management is not possible in many cases.

Although studies have been conducted on sharp injuries in developing countries [9, 10], there are few available data on sharp injuries and reporting behavior in China. To understand the risk factors for sharp injuries and determine the corresponding preventive countermeasures, we carried out a cross-sectional investigation at a provincial teaching hospital in Anhui province, China.

## Methods

The study was carried out in a tertiary affiliated teaching hospital of medical college in Anhui province, China, in February 2017. We investigated 901 HCWs from January to December 2016, using a self-administered questionnaire. This hospital is a provincial teaching hospital which has 1050 beds and employs approximately 1200 HCWs. The questionnaire was based on sharp injuries in China and other countries [11–13]. A pre-survey was conducted in the surgery department on December 20 2016. We distributed 36 questionnaires and 34 were available for analysis, resulting in an overall response rate of 94.4%. According to the pre-survey and existing problems, the questionnaire was revised by a group of professors and members of the infection control committee of the hospital who confirmed the validity of its content.

The questionnaire consisted of three parts. The first part was designed to obtain personal information on the HCWs, such as job category, gender, education, and department. The second part sought to gather information on factors associated with sharp injuries in the past year. The third part focused on the reporting rate of sharp injuries. The completed questionnaires were entered into Epi Info™ and analyzed using SPSS version 20.0. The association between independent variables and exposure were evaluated using an independent sample Student *t* test and chi-square test. Stepwise logistical regression was used to analyze the risk factors. A *P* value of < 0.05 was considered statistically significant.

All participants were fully informed regarding the purpose of the study, and informed consent was obtained from each participant. The collected data were treated as confidential.

## Results

### Characteristics of the included HCWs

Of 1000 HCWs invited to participate in this study, 901 returned completed survey forms. The overall response rate was 90.1%, and included 301 doctors and 600 nurses (227 males and 674 females). Among the participants, 129 (14.3%) HCWs had worked in the hospital >20 years. Some 363 (40.3%), 341 (37.8%) and 197 (21.9%) HCWs had a college, bachelor, or master degree, respectively. More than half of HCWs (57.7%) had a primary title with professional qualifications. Almost 30% of HCWs (29.7%) had an intermediate title, and 113 (12.5%) held the title of professor. Most of the HCWs were from the department of surgery 371 (41.2%) and internal medicine 311 (34.5%). The number of HCWs who often attended training programs was only 388 (43.1%), and 677 (75.1%) had standard infection prevention knowledge (Table 1).

**Table 1:** Characteristics of the included HCWs (*N* = 901)

Characteristics		Number	Constituent ratio (%)
Gender	Male	227	25.2
	Female	674	74.8
Seniority	< 5 years	393	43.6
	5~20 years	379	42.1
	> 20 years	129	14.3
Job category	Doctor	301	33.4
	Nurse	600	66.6
Title	Primary	520	57.7
	Intermediate	268	29.7
	Professor	113	12.5
Education	College	363	40.3
	Bachelor	341	37.8
	Master	197	21.9
Department	Surgery Dept.	371	41.2
	Internal medicine	311	34.5
	Obstetrics and Gynecology	35	3.9
	Emergency Dept.	72	8.0
	Infection Dept.	25	2.8
	Operation room	73	8.1
	Sterile supply room	14	1.6
Training programs	Seldom	513	56.9
	Often	388	43.1
Standard prevention Knowledge	Unknown	224	24.9
	Known	677	75.1

### Factors associated with the occurrence of sharp injuries

We analyzed the factors associated with the occurrence of sharp injuries using the chi-square test, which showed that factors such as seniority, job category, title, education, department, and training more or less had an effect on the occurrence of sharp injuries (*P* < 0.05). The incidence of sharp injuries among nurses (31.2%) was higher than that among doctors (19.9%). However, there was no statistically significant difference between male and female participants (*P* = 0.103). HCWs who seldom attended training programs were more prone to sharp injuries (33.3%) than those who often attended training programs (19.6%). A chi-square test between two similar variables showed that the incidence of sharp injuries among HCWs whose seniority was < 5 years (34.4%) was higher than those whose seniority was > 5 years (*P* < 0.05). Similarly, there were statistically significant differences between the primary title and other titles (*P* < 0.05), and the incidence of sharp injuries among HCWs with a junior college degree (35.3%) was statistically higher than those with a bachelor or master degree (*P* < 0.05). In addition, HCWs in obstetrics and gynecology were more likely to have sharp injuries (42.9%) than HCWs in other departments according to our study (*P* < 0.05) (Table 2).

**Table 2:** Single factor analysis of factors associated with the occurrence of sharp injuries (*N* = 901)

Variables		Number (*N* = 901)	Sharp injuries		χ^2^	*P* value
			Number	Incidence (%)		
Gender	Male	227	53	23.3	2.65	0.103
	Female	674	195	28.9		
Seniority	< 5 years	393	135	34.4	17.97	0.000
	5~20 years	379	90	23.7		
	> 20 years	129	23	17.8		
Job category	Doctor	301	61	19.9	11.94	0.001
	Nurse	600	187	31.2		
Title	Primary	520	173	33.3	21.23	0.000
	Intermediate	268	49	18.3		
	Professor	113	26	23.0		
Education	Junior college	363	128	35.3	18.24	0.000
	Bachelor	341	76	22.3		
	Master	197	44	22.3		
Department	Surgery Dept.	371	125	33.7	25.55	0.000
	Internal medicine	311	60	19.3		
	Obstetrics and Gynecology	35	15	42.9		
	Emergency Dept.	72	19	26.4		
	Infection Dept.	25	10	40.0		
	Operation Room	73	15	20.5		
	Sterile supply room	14	4	28.6		
Training programs	Seldom	513	172	33.3	21.52	0.000
	Often	388	76	19.6		

Using stepwise logistic regression analysis, the occurrence of sharp injuries was included as the dependent variable, while job category, seniority, title, training programs, education, and department were independent variables. We found that seniority and training programs were the risk factors associated with the occurrence of sharp injuries. HCWs whose seniority was > 20 years were associated with a decrease in risk for the occurrence of sharp injuries. However, HCWs who seldom attended training programs were associated with an increase in risk for the occurrence of sharp injuries (Table 3).

**Table 3:** Stepwise logistic regression analysis of factors associated with the occurrence of sharp injuries

Variables	OR	95% CI
Seniority		
< 5 years		
5~20 years	1.3	(0.5, 3.3)
> 20 years	0.3	(0.1, 0.9)
Training programs		
Often	1	
Seldom	5.4	(2.4, 12.3)

### Reporting rate and reasons for underreporting of sharp injuries exposed to blood

Of the 130 respondents who experienced sharp injuries exposed to blood, only 44 HCWs (33.9%) reported their injuries to the concerned body. There was a statistically significant difference between the type of blood exposure and injury level (*P* < 0.05). However, there were no significant differences between gender, job category, activity, and department (*P* > 0.05) (Table 4).

**Table 4:** Reporting rate of sharp injuries exposed to blood

Variables		Number of sharp injuries exposed to blood (*n* = 130)	Reporting rate of sharp injuries exposed to blood		*χ* ^2^	*P* value
			Reporting number	Reporting rate (%)		
Gender	Male	31	12	38.7	0.43	> 0.05
	Female	99	32	32.3		
Job category	Doctor	55	23	41.8	2.71	> 0.05
	Nurse	75	21	28.0		
Activity	Disposing used medical waste	8	4	50.0	8.30	> 0.05
	During operation	23	11	47.8		
	Blood drawing	21	9	42.9		
	During medical examination	16	6	37.5		
	Destroyed sharps items	19	6	31.6		
	Withdrawing the needle	43	8	18.6		
Department	Surgery Dept.	67	22	32.8^**^	20.29^※^	< 0.01
	Internal medicine	35	9	25.7^**^		
	Emergency Dept.	6	4	4/6		
	Infection Dept.	7	7	7/7		
	Pediatrics	15	2	13.3^**△^		
Type of blood exposure	HBV	45	33	73.3^##^	65.40^※^	< 0.01
	HCV	5	5^◇^	5/5^##^		
	Unknown	80	6	7.5		
Injury level	Light	52	10	19.2	12.60^※^	< 0.01
	Medium	75	31	41.3		
	Heavy	3	3	3/3		

^◇^included 1 case of HIV exposure

^※^For rank sum test and two rank and inspection, compared with infection Dept

^**^P < 0.01; Compared with the emergency department

△*P* < 0.05; Compared with the unknown exposure

^##^*P* < 0.01

The main reasons for not reporting the sharp injuries were as follows: perception that the extent of the injury was light (30.2%), having antibodies (27.9%), and unaware of injury (16.3%). Other related reasons for not reporting were unfamiliar with the reporting procedures (11.6%), thinking that the injury was due to improper operation by themselves (7.0%), and too busy working to report (7.0%). There was a significant difference in the unreported reasons between doctors and nurses (*P* < 0.05). The main reasons for not reporting in doctors were as follows: thinking that the extent of the injury was light (53.1%) and having antibodies (21.9%), while the main reasons for not reporting in nurses were thinking that they had antibodies (31.5%) and unaware of injury (24.1%) (Table 5).

**Table 5:** Reasons for underreporting of different job category

Job catagory	*n*	Reasons for underreporting						*χ* ^2^	*P* value
		Perception that the injury is light	Having antibodies	Unaware of injury	Unfamiliar with the reporting procedures	Improper operation	Too busy working to report		
Doctor	32	17 (53.1)	7 (21.9)	1 (3.1)	2 (6.3)	3 (9.4)	2 (6.3)	2.73	< 0.01
Nurse	54	9 (16.7)	17 (31.5)	13 (24.1)	8 (14.8)	3 (5.6)	4 (7.4)		
Total	86	26 (30.2)	24 (27.9)	14 (16.3)	10 (11.6)	6 (7.0)	6 (7.0)		

## Discussion

Of 1000 HCWs invited to participate in this study, 901 returned completed survey forms, resulting in an overall response rate of 90.1%. The incidence of sharp injuries was 27.5%, which was similar to the incidence reported in other studies [14, 15]. Of 248 sharp injuries, 130 HCWs were exposed to blood. HCWs are vulnerable to accidental exposure to blood and other body fluids while performing clinical activities [16]. Sharp injuries mainly take place during the disposal of used medical waste, and can lead to the transmission of pathogens. At least 20 different pathogens including viruses, bacteria, and fungi can be transmitted to HCWs through sharp injuries. The most common blood-borne diseases are HBV, HCV, and HIV. A national seroepidemiologic survey in 1992 found that the carriage of HBV surface antigen was 9.8% in China. In our study, HBV surface antigen-positive HCWs accounted for 73.3%, which demonstrates that the prevalence of chronic HBV infection is high in China. However, we observed no seroconversions in 44 HCWs who reported sharp injuries, confirming that the risk of transmission of blood-borne viruses in these events is very low; however, these events should still be a focus of attention.

The present study found that nurses had a significantly higher frequency of occupational sharp injuries than doctors. Nurses have more chance of contact with sharp instruments, such as needles, when administering injections to agitated patients and disposing of sharp instruments [17]. Nurses often feel tired due to lack of sleep [18], and have varying degrees of job burnout leading to adverse psychological reactions. Sharp injuries further increase job burnout, which lead to a vicious cycle [19]. Sharp injuries often occur in HCWs whose seniority is of short duration. This may be the result of careless work, unskilled technology, nonstandard operations, and poor understanding of occupational safety [20]. The number of HCWs with a primary title injured by sharp instruments was significantly higher than that with a high title. The study also found that different departments had different incidence rates of sharp injuries. The highest number of sharp injuries occurred in the surgery department, and may be related to the characteristics of this department, such as more invasive operations and time constraints. Furthermore, HCWs who seldom attended training programs were more likely to be injured by sharp instruments. There is growing evidence that many cases of occupational exposure to blood and body fluids through needle-stick and sharps injuries are unreported [5, 21].

In our study, the reporting rate of sharp injuries was 33.9% (44/130), and reporting of these injuries varied significantly in different departments. The department of infectious diseases reported all incidents of sharp injuries. HCWs in this department were frequently in contact with patients with blood-borne infectious diseases, attached great importance to occupational safety, and also paid greater attention to sharp injuries compared with other departments. Different sources of exposure had a different reporting rate. When the source of exposure was HBV, the level of concern in HCWs decreased significantly. This is because China has universal HBV vaccination, and most HCWs had antibodies. When the exposure source was HCV or HIV, which have no effective drugs for prevention or treatment, HCWs paid particular attention and a report was prepared when occupational exposure occurred. When the infection source was unknown, HCWs may take measures themselves without preparing a report. In addition, the reporting rate varied with different degrees of injury. Without sufficient understanding of self-protection, HCWs considered that minor injuries had a low probability of infection, so they paid little attention to these injuries, especially minor injuries, which was one of the main reasons for the lack of reporting in clinical doctors. Some HCWs believed that occupational infections were unlikely to happen to them, and so paid little attention to reporting them. Some HCWs did not know how to report the injury to the concerned body. This may be due to an incomplete reporting procedure, poor information, insufficient recognition of the damage caused by blood-borne occupational diseases, and inappropriate treatment after reporting. Some HCWs considered that sharp injuries were caused by themselves and were afraid of being criticized, so they refused to report the incident.

Although injuries due to sharp instruments in HCWs are inevitable, evaluations by the United States Centers for Disease Control and Prevention showed that 62–80% of sharp injuries can be prevented [22]. As 22–52% of needle-stick injuries are the result of recapping, attempts to reduce the incidence of needle-stick injuries have focused on eliminating such practices [23, 24]. However, many approaches such as recommendations against recapping and increasing the accessibility of disposal containers have failed to reduce the incidence of needle-stick injuries [25]. Froom et al. proposed that a lecture stressing the dangers of needle-sticks and recommending the use of the scooping method was effective in reducing the risk of needle-stick injuries in medical students [26]. In this hospital, we carried out several preventive countermeasures, such as equipping HCWs with the necessary personal protective equipment, wearing gloves before contact with a patient’s blood and body fluids, a ban on recapping needles after use, eliminating unnecessary injections, using automatically retracting safety syringes, and disposing of sharps into a sharps container immediately after use. Although we issued relevant documents on occupational safety, poor compliance with standard precautions is still a risk factor for sharp injuries. The training program on occupational safety requires strengthening, especially for junior HCWs. An effective training program is essential, as its implementation will decrease the overall rate of sharp injuries [27, 28]. The most common action taken after sharp injuries are compression, washing the site of injury with water and soap, and taking post-exposure prophylaxis against HBV and HIV after injury. After exposure to blood, it is necessary to check immunology markers of HBV, HCV, HIV, and other blood-borne diseases, set up health records and provide a reference for prevention.

## Conclusions

This study attempted to investigate the incidence and reporting behavior of sharp injuries and to identify factors associated with sharp injuries among HCWs in Anhui province, China. Our study provided evidence that sharp injuries in the studied hospital were common and were likely to be underreported. Therefore, an effective reporting system and sufficient education on occupational safety are needed in the relevant institutions. Moreover, it is important to take effective measures to manage sharp injuries among HCWs and provide guidance for prevention.

## Abbreviations

HBV: Hepatitis B virus
HCV: Hepatitis C virus
HCWs: Healthcare workers
HIV: Human immunodeficiency virus
